# Jottings

**Published:** 1909-11-20

**Authors:** 


### Jottings.

The Radcliffe-on-Trent (Notts) County Council has appointed a sub-committee to consider plans and details relating to the enlargement of the county asylum.

The Princess of Wales paid an informal visit to King's Lynn on Thursday last and honoured with her presence and support the Art Loan Exhibition which is being held at the St. James's Hall to benefit the funds of the West Norfolk and Lynn Hospital.

At the last quarterly meeting of Governors of the Royal Devon and Exeter Hospital it was pointed out that the decrease of income, which for the nine months amounted to £3,167, is rather serious. The figures show that the Governors can not meet their liabilities by the subscriptions of the benevolent public, and they are always under water. The hospital is economically managed, and there is no way of retrenching expenditure except by closing a ward, which would inflict an injury on the public.

The annual report of Chesham Cottage Hospital for the year ending June 30, 1909, shows a deficit of £28—less than that of the year before. The report goes on to say that "never before in the history of the hospital has better work been performed than at the present time." Local efforts are being made vigorously to raise what funds are needed.

The proposal that the Poor Law unions of Surrey should unite in establishing an institution for the treatment of the phthisis cases of the county chargeable to the rates has practically fallen through. The Croydon Guardians, who were the conveners of the recent conference on the subject, received letters from the Chertsey, Dorking, Epsom, Farnham, Guildford, Reigate, and Richmond Boards of Guardians declining to take further action.

Sir Titus Salt's Hospital, Saltaire, was reopened on the 2nd inst., after extensive alterations.

At the annual meeting of the Birmingham Orthopædic and Special Hospital the Chairman reported that the subscriptions had fallen off to such an extent that it had been necessary to place donations to current account. One subscriber wrote explaining that the cessation of his subscription was due to the Budget.

Mr. F. Wadham Elers, M.A., J.P., for some 13 years Chairman and Treasurer of the General Hospital at Tunbridge Wells, and most active in the philanthropic and public affairs of the town, has, at the unanimous wish of the Town Council, accepted the position of Mayor of the Borough for the coming year.

The King sent a number of pictures to the art loan exhibition opened in Lynn on Friday on behalf of the West Norfolk and Lynn Hospital. The Queen has sent exhibits from the art schools at Sandringham, and the Princess of Wales has lent several articles. The Prince of Wales has sent a cheque for £50 towards the funds of the hospital. His Royal Highness is lending thirteen silver models of ships.

The Fresh Air Fund in connection with the Ulster Hospital for Children, Mount Pottinger, Belfast, issues its annual appeal. Through this fund three beds are retained in the Homes of Rest, Bangor, for suitable cases from among the children treated in the wards. Contributions will be gratefully acknowledged by any member of the staff, or they may be sent to Miss Tate, lady superintendent, at the hospital.

